# "I Cannot Be Worried": Living with Chagas Disease in Tropical Bolivia

**DOI:** 10.1371/journal.pntd.0005251

**Published:** 2017-01-18

**Authors:** Colin J. Forsyth

**Affiliations:** Department of Anthropology University of South Florida 4202 E. Fowler Avenue, SOC 107 Tampa, FL, United States of America; Walter and Eliza Hall Institute, AUSTRALIA

## Abstract

**Background:**

Chagas disease (CD) profoundly affects the social and emotional dimensions of patients’ lives, and disproportionately impacts poor, marginalized populations in Latin America. Biomedical treatment for CD fails to reach up to 99% of the people affected, and in any case seldom addresses the emotional health or socioeconomic conditions of patients. This study examines patient strategies for coping with CD in the department of Santa Cruz, Bolivia.

**Methodology:**

In this ethnographic study, semistructured interviews took place from March-June 2013 with 63 patients who had previously tested positive for CD. During the fieldwork period, participant observation was conducted and patient family members, providers, community members, and public health officials were consulted.

**Principal Findings:**

Patients often experienced emotional distress when diagnosed with CD, yet were generally unable to find biomedical treatment. Respondents stressed the need to avoid powerful emotions which would worsen the impact of CD symptoms. To manage CD, patients embraced a calm state of mind, described in Spanish as *tranquilidad*, which partially empowered them to return to a normal existence.

**Conclusions:**

In the perceived absence of biomedical treatment options, patients seek their own means of coping with CD diagnosis. Rather than fatalism or resignation, patients’ emphasis on maintaining calm and not worrying about CD represents a pragmatic strategy for restoring a sense of normalcy and control to their lives. Programs focused on treatment of CD should remain mindful of the emotional and social impact of the disease on patients.

## Author Summary

An estimated 99% of people with Chagas disease do not receive treatment. I interviewed 63 patients with a diagnosis of Chagas disease at a rural clinic in an endemic area of Bolivia. Only 4 had obtained biomedical treatment. Although the departmental government provided free medication for Chagas disease, this was not typically available for adults in patients’ communities, and additional costs such as consultations and cardiologic examinations were prohibitively expensive for this marginalized population. Travel distances and contradictory messages from providers also made it difficult for patients to access treatment. Furthermore, patients believed strong emotions would worsen the symptoms of their Chagas disease, yet often felt anguished or worried when diagnosed. To deal with the emotional impact of the disease and restore normalcy to their lives, patients adopted a strategy of remaining calm and minimizing worries about Chagas disease. This is not fatalism or resignation, but a pragmatic strategy patients utilize to feel more in control of the disease. Treatment programs need to consider the emotional and social implications of Chagas disease in addition to addressing structural barriers which impede treatment.

## Introduction

The consequences of disease transcend the realm of the biological, impacting not only the physical functioning of the human body, but the social life and emotional wellbeing of the affected person. While providers focus on treating the clinical aspects of disease, patients deal with multiple levels of impacts, particularly emotional and socioeconomic ramifications. This study investigates how people with Chagas disease (CD) in an endemic area of Bolivia cope with their diagnosis in order to continue functioning in their daily lives.

More than six million people worldwide are infected with *Trypanosoma cruzi*, the protozoan which causes Chagas disease (CD)[1–3]. In Latin America, where it is endemic, CD creates a greater burden in years of productive life lost than all other parasitic infections combined[4]. *T*. *cruzi* is typically spread by hematophagous triatomine vectors, but blood transfusion, organ transplant, congenital transmission and oral ingestion are viable routes of infection[5]. There is an initial acute phase with highly variable symptoms, followed by an asymptomatic indeterminate phase. Years or decades after the initial infection, 30–50% of people in the indeterminate phase progress to a chronic stage of CD[6, 7], which is usually characterized by cardiomyopathy, although gastrointestinal complications including megaesophagus and megacolon affect many patients, especially in the Southern Cone of South America[5].

CD, like other neglected tropical diseases (NTDs), is intrinsically linked to socioeconomic inequalities, which drive rates of infection[8]. The epidemiology of CD has been profoundly influenced by historical patterns of marginalization and exploitation in Latin America, and the disease continues to disproportionately affect the poor in both endemic and non-endemic settings[9, 10]. CD and other NTDs are highly stigmatized due to their association with poverty and rurality[11]. NTDs have received scant attention from pharmaceutical research and development, and thus medications tend to be outdated and ineffective[12, 13]. For example, the only two medications for treating CD, benznidazole and nifurtimox, were developed over 40 years ago, produce frequent side effects, and are less effective in treating adult patients[14].

### Treatment of Chagas Disease in the Bolivian Health System

Bolivia has the highest prevalence of CD in the world, with at least 6% of the population affected[1]. More than 99% of people with CD in Bolivia and worldwide are undiagnosed and untreated[15–17]. It was previously thought that treatment of adults with chronic CD was ineffective, and that complications from CD were due to an overactive immune response[18]. However, Argentinian studies in the mid-2000s demonstrated lower mortality in adult patients with chronic CD who received antiparasitic treatment[19, 20]. Shortly thereafter, *Médecins sans Frontières*/Doctors without Borders began treating adult patients in the department of Cochabamba, and the Pan American Health Organization’s 2010 Chagas strategy asserted that “care of infected adults—should be a guaranteed part of primary care”[21]. Nonetheless, many Bolivian doctors still operate under the assumption that CD should not be treated in adults[22].

In 2006, newly elected President Evo Morales signed the National Chagas Law, which made elimination of the disease a “national priority”[23]. In 2008, Bolivia’s National Chagas Program began developing pilot programs to treat adults in Cochabamba, Tarija and Potosí[24]. In 2011, Santa Cruz’s departmental Chagas program began offering treatment to adults via three clinics and two hospitals in the departmental capital[25]. In an effort to promote accessibility, the program provides free medication and consultations to patients, although patients still have to pay for some laboratory analyses and an examination with a cardiologist. Moreover, this treatment is extremely difficult for patients in rural areas and smaller communities to access. These patients often perceive there is no viable treatment option, forcing them to simply accept the disease as an unalterable aspect of their lives. Meanwhile, they deal with the considerable emotional toll and uncertainty of living with a potentially fatal infection. This study examines how patients in an endemic area of Bolivia cope with the social and emotional impact of CD,

## Methods

In this ethnographic study, a convenience sample of 63 adults with a positive CD diagnosis were interviewed. All were patients of the Centro Medico Humberto Parra (CMHP) in Palacios, Bolivia, a nonprofit clinic which provides free or low cost service to several communities north of Santa Cruz, Bolivia’s largest city. Palacios is a rural community where most work as agricultural labors, earning $40 monthly in 2001 ($54.56 in 2016 dollars)[26]. A review of the clinic’s screening records showed a CD prevalence of 57%. Locally, no treatment for CD is available, and most patients are unaware that treatment exists in urban hospitals, which in any case requires lengthy travel. During the fieldwork period, in addition to patients, the author spoke with clinicians, public health officials, health promoters, and patient family members.

Interview questionnaires were developed based on two theoretical orientations in medical anthropology. First, Kleinman’s explanatory models framework was utilized[27]. Kleinman argues that both patients and healthcare personnel develop individual explanatory models to interpret and address each healthcare episode. Each individual’s explanatory model encompasses five dimensions: etiology, timing of symptoms onset, pathophysiology, course of sickness, and treatment options. Kleinman also emphasizes the distinction between “illness” and “disease”[28]. Providers focus on disease, the clinical and biological aspects of a particular health condition. However, patients deal with illness, a broader term which also takes into account the social and emotional ramifications of health concerns. Kleinman envisions the explanatory models concept as a tool to improve patient-provider communication[27].

However, the explanatory models framework has been criticized for inattention to the structural determinants of health[29, 30]. Farmer, like Kleinman, urges researchers to gather “illness narratives,” or individuals’ descriptions of healthcare episodes[31]. Yet Farmer also emphasizes the need for analysis of how inequalities in the social and political environment shape access to healthcare and impact epidemiology[32]. Farmer refers to the disproportionate burden of morbidity and mortality among the world’s poor as “structural violence.” This concept stresses that inequalities in burdens of disease and access to healthcare can kill just as surely as overt physical violence. For instance, Farmer describes how the marginalization and disenfranchisement of the poor, largely due to global economic processes, has driven the spread of AIDs and tuberculosis, not only in underdeveloped countries such as Haiti, but among disadvantaged populations in wealthier nations[33].

For this study, questionnaires are primarily based on Kleinman’s explanatory models concept. Patients were asked what they felt were the causes and symptoms of Chagas disease, as well as what treatment strategies they utilized. Each patient’s “illness narrative,” or description of their experience with CD, was solicited. However, other questions focused on patients’ interactions with the healthcare system in order to assess the impact of structural inequalities in their experience of managing CD.

Data collection took place from March-June 2013. While serving as a volunteer at the CMHP providing CD education, the author recruited patients for the study. Patients were recruited when they waited for services at the CMHP. Two people refused to participate. All 63 patients who agreed to participate self-reported a previous diagnosis of CD. The author is fluent in Spanish and had previously lived in the study area. Although no pilot was performed for the semi-structured interviews, questionnaires were developed based on an extensive literature review which focused on CD as well as medical anthropological literature pertinent to the study area. Questions were reviewed by a committee of anthropologists at the University of South Florida and by Bolivian staff at the CMHP. Semi-structured interviews were conducted by the author in Spanish, either at the CMHP (n = 44) or in patients’ communities (n = 19). Interviews were recorded and transcribed in Word, then analyzed and coded for themes in Atlas.ti, using a grounded theory approach[34].

### Ethical Statement

The study received ethical approval from the University of South Florida Institutional Review Board (Approval # Pro00010734), as well as an ethical review board at the CMHP. Because the patient population is highly marginalized, has low access to education, and in some cases speaks Spanish as a second language, the IRB granted a waiver of written consent and approved the use of a verbal informed consent script. Recording of interviews only commenced after participants provided verbal informed consent, which was documented by the researcher. Respondents’ names have been changed to protect their anonymity.

## Results

Most patients (n = 49, 78%) were female; which mirrors the patient population of the CMHP. Patient age ranged from 29–82 years old, and the median age was 54. The majority of patients in the sample were socially and economically marginalized. While 63% had received less than a primary level of education (Table 1), nearly half (48%) reported earning less than the Bolivian minimum wage. The bulk of respondents (n = 50, 79%) were from the department of Santa Cruz, although some had migrated from other parts of Bolivia. Most patients were initially diagnosed in the CMHP (n = 25, 39.6%) or blood banks (n = 11, 17.5%); the rest found out from doctors, hospitals, or employers.

**Table 1:** Sample Characteristics.

		n	%
**Gender**	Male	14	22
	Female	49	78
**Age**	29–39	7	11
	40–49	16	25
	50–59	23	37
	60–69	14	22
	70–82	3	5
**Education**	Primary or less	40	63
	> Primary	23	37
**Treated for Chagas**	Yes	4	6
	No	59	94
**Department of Origin**	Santa Cruz	50	79
	Other	13	21
**Community**	Buena Vista	8	13
	Minero	9	14
	Palacios	5	8
	Warnes	12	19
	Yapacaní	8	13
	Other	21	33

### Impact of Diagnosis

Respondents were asked how they felt when they were diagnosed with the disease. Twenty (31.7%) replied that they felt frightened, worried or emotionally devastated. “I felt bad,” said Jacinta, “it affected me a lot to know I had that disease. I didn’t eat, I didn’t sleep. I felt bad, thinking that I was going to die.” When Betty, a 46-year-old homemaker, found out she had CD, she cried and “became frantic. It was the saddest thing, to have Chagas, because they said it was dangerous, that you died from it.” Constanza, a 62-year-old supervisor, described becoming upset, “because I have family with Chagas. And of course, when they said I have Chagas, and at any moment I could die, of course I became sad. I thought a lot about the disease. I worried a lot.” Constanza’s sadness and worry stems from the fear that her life could end unexpectedly.

People who receive a diagnosis of Chagas must learn to cope with the knowledge that at any moment their lives could end. This feeling is evident in the words of Daniel from Yapacaní:

There are a lot of times when it worries you, right? One, for the work you do. You have the thought that at any moment you can end up dead. And all the work you are doing might be for nothing.

Daniel, an otherwise healthy 39-year-old father and agricultural worker, was forced to confront the possibility of death following his diagnosis of CD.

### Difficulties in Accessing Treatment

In Bolivia’s public health system, providers are classified into three levels. Level I facilities provide basic care, often via a nurse or community health worker, and are more readily available in smaller communities. These constitute more than 90% of health facilities in the public system[35]. Level II facilities are similar to clinics or community hospitals in the United States, and are able to provide services such as surgery or internal medicine. Level III facilities are the largest hospitals, treat the full range of health issues, and are generally only located in urban areas. In the study area, only the city of Santa Cruz, 90 km distant from the CMHP, had level III facilities where patients with complications from advanced chronic Chagas disease could receive surgeries or other necessary interventions. Anti-parasitic treatment for CD was not available in the level I clinics in patients’ communities, whereas the level II hospital in Warnes only treated children under 15.

Only 4/63 patients (6.3%) had received antitrypanosomal treatment for their CD, all via private or semi-private providers. No patients had received free medication through the Departmental Chagas Program; all but 2 lived in communities where this option was unavailable. None of the respondents were aware that free treatment for adults with CD existed. Patients, providers and public health officials described several barriers to diagnosis and treatment of CD. The main barrier to diagnosis was the lack of confirmatory testing. The ELISA required to confirm diagnosis of CD and initiate treatment was only available in larger hospitals in urban centers (referred to as Level II and III centers). Level I facilities, which provide primary care in smaller communities, did not always have supplies available even to perform initial screening for CD. Obtaining the confirmatory diagnosis required sending a sample to an urban level II or III hospital with ELISA capability and waiting up to several weeks for results. Moreover, patients sometimes experienced confusion about the purpose of laboratory testing. While clinicians used testing simply to detect the presence of *T*. *cruzi* antibodies, many patients expected the results to indicate if their CD had progressed to an advanced phase. This sometimes caused patients who already had a positive diagnosis to request unnecessary repeat testing with rapid assays which were unable to provide information about the progression of the disease.

The main barriers to treatment were distance, indirect costs such as laboratory analyses, transportation and missed work, and contradictory information from providers about the benefits of antiparasitic treatment for CD. Patients had to travel substantial distances to reach the few hospitals and clinics in the departmental capital which offered treatment for CD[36], which entailed additional expenses and missed work. As the following quotes illustrate, patients were apprehensive about accessing biomedical care for fear of incurring heavy costs.

The truth is, our resources just aren’t enough to go to the doctor and do analyses. No, it’s expensive, Santa Cruz is very expensive… So what poor person is going to go see the doctor, get checked by a doctor…? Because what they have is basically just for food. Because you earn very little… and sometimes there’s not enough money. Many people live like this. (Alma, 55, homemaker)

They did all these tests, but they never gave me any treatment, not one tablet, nothing. No, but that is the last time that I go to the doctor, because the truth is, well, you just pay and you don’t get better. (Nora, 48, storekeeper)

They don’t attend you for a long while. Until you are practically dead… If I go, I lose the whole day. And sometimes you don’t have any days off… It’s a lot of cash you have to spend there. Everything is money. (Mario, 51, night watchman)

All three of these patients perceived that, after considerable investments of time and money, they had been unable to make headway in their efforts to seek medical care for CD. They lacked the resources to continue to make efforts to seek care, and were not aware of the existence of free antiparasitic treatment through the departmental Chagas program. The lack of perceived treatment options compounded the emotional impact of diagnosis; patients in this study felt they were living with a deadly disease for which there was no medical recourse.

### Learning to Live with Chagas-the Notion of “Tranquilidad”

Upon perceiving that treatment for their CD was unavailable or difficult/impossible to access, patients sought alternative means of maintaining their health and continuing with their normal lives. Many made use of ethnomedical treatments in an effort to cure their CD or manage its symptoms[37]. Most patients also conveyed a sense of acceptance and peace with their diagnosis, affirming that it did not worry or upset them. One of the most frequently coded themes in the interview transcripts, with 54 related quotations, was the concept of *tranquilidad*. Literally, this translates to calm, peacefulness, or contentment. Patients with CD consider *tranquilidad* a desirable state of consciousness. In part, this is because freeing the mind of worry allows them to continue with their daily lives.

As the following quotes illustrate, patients feel keeping emotions calm and maintaining a state of *tranquilidad* is critical for preventing a worsening of CD symptoms:

Foremost is being *tranquila* [calm], no? Being *tranquila*. Not thinking that you are sick. Getting your mind focused on some other thing, right? Because if you constantly worry about the disease, it becomes worse. (Pura, 67, homemaker)

I have been told that I cannot be troubled. I cannot be worried. And if someone is arguing or there is some kind of problem, you have to get away from it, they say. If not, you are going to get palpitations. (Hortensia, 54, homemaker)

We have to calm down (*tranquilizarnos*), because if we become upset, it is another ill at the same time. (Betty, 46, homemaker)

The first thing, you know what it is? One’s *tranquilidad*. That’s right. Because that way, the heart is not much affected, you don’t get palpitations. *Tranquilidad* is the main thing, because there are times when you get palpitations, no? (Constanza, 62, supervisor)

These examples underscore the need to be calm (*tranquilo*) not only for one’s emotional wellbeing, but to lessen the effects of CD. Even otherwise positive emotions might be destructive if they impact the *tranquilidad* of a person with CD. “I can’t get excited,” says Pura. “I avoid anything like that. I can’t go to parties. Sometimes I get mad, I get really happy, and all that does damage. It does damage, so you have to be just normal.”

Ten of the twenty respondents who initially felt upset upon receiving a diagnosis of Chagas describe a shift where they take control of their feelings and become *tranquilos*. “I felt so bad, thinking that I was going to die,” says Jacinta, a 61-year-old homemaker. “It affected me a lot. So then, I asked the Lord and the Virgin to take those thoughts away. And I got rid of them, and I forget that I have Chagas.” According to Doris, a 60 year-old who ran a small restaurant out of her home, “When a person gets the disease, a lot of the times it’s psychological. Because when I first found out I wept. ‘How could I get sick with [Chagas], and I’m not even 30?’ Then I said, bah, and I lived my life and I forgot about it.” In Betty’s case, talking to a friend helped her overcome the initial anguish over her diagnosis: “…my friend told me, ‘don’t worry because all of us have Chagas. I have Chagas and look at me.’ From that I felt a little more *tranquila*.” In each instance, the individual overcame worry about CD, while emphasizing that this helped them live their lives normally.

In three other cases, respondents describe doctors reassuring them and calming them when giving the diagnosis. If their disease is latent and asymptomatic, doctors often tell them their CD is sleeping. “You have Chagas, but it’s no cause for alarm,” the doctor told Elisa, 44. “Don’t worry, it’s asleep,” the doctor told Yoana, 64. When Fernanda, a 57 year-old homemaker received her initial diagnosis, she felt “really scared. They told me, ‘No, no, no. It’s asleep.’ So I calmed down.” In these examples, practitioners are concerned with ensuring patients feel calm upon receiving their diagnosis.

Thirty participants (47.6%) affirmed that they felt calm–*tranquilo*–and did not think or worry about their Chagas disease. Alma likens the state of *tranquilidad* to not having Chagas at all.

Until now I feel that I don’t have Chagas. *Tranquila*, I don’t feel palpitations in my heart. I think that–it’s like I don’t have the disease, no? So, I feel *tranquila* because others say they have Chagas and feel agitation in their hearts.

In part, the peace she feels is due to her lack of symptoms, but just as importantly, she does not permit herself to worry about the possibility of developing a more severe, symptomatic form of the disease. Alma perceives that such worrying would act as a trigger that aggravates her CD. In a similar example, Porfiria, 54, indicates her diagnosis of CD “didn’t affect me… I didn’t say, ‘Oh no, it’s going to kill me,’ or anything. Well, you’ve got Chagas. *Tranquila*, nothing more. As if it were nothing.”

Importantly, t*ranquilidad* implies acceptance of the possibility of death which a diagnosis of CD entails. Hugo, a 62-year-old teacher, avows he feels, “*Tranquilo*. Because we have arrived in this world, and I am conscious that we have to depart as well. It’s not forever. *Tranquilo*. *Tranquilo*.” Elsa, a 35-year-old storekeeper, also considers herself “*tranquila*. Anyway, you’re going to die of something. You just have to wait for it.” Josefina, 53, who works cleaning houses, asks, “Why are we going to get upset? *Tranquilo*, you have to die from something.”

Five respondents (7.9%) mentioned faith in God or the Virgin helps bring about acceptance of the disease and *tranquilidad*.

I was sad a long time. I told my children and my husband that I had this disease, and they told me you have to have faith that God will protect us and care for us. From then on we always ask God and the Virgin to protect us and care for our health. (Leticia, 47, homemaker)

Constanza “worried a lot” when she first found out she had Chagas, but later came to accept it, something she attributes to “God. Because God comes first, you see? And that gave me more courage with this disease.” In all these examples, faith helps individuals come to terms with having the disease, and in particular enables them to put an end to worrying.

Two people mentioned they use work as a means of putting their minds at ease. “I felt *tranquilo*, even though I had Chagas, I didn’t give it a lot of importance,” says Eduardo, a 54-year-old agricultural worker. “That was the good thing.” When asked what helped him feel calm, Eduardo responded, “being able to work, do something, clean or straighten up, I feel happy. And well, you get used to it.” “They told me it was better not to worry about the disease that I had,” recalls Anita, a 47-year-old vendor of *salteñas*, a local delicacy similar to an empanada. “I know it can get worse very quickly. I spent my whole life working, and in that I keep things normal. It’s as if I didn’t have the disease. I work and work and work, and I don’t even know if I’m sick or anything.” For these individuals, being able to continue functioning normally is the key to *tranquilidad*. At the same time, these patients perceive it is essential not to become too worried or upset, since this might set off the symptoms of Chagas disease.

It would be incorrect to state that *tranquilidad* stems from avoidance of dealing with CD. In an illustrative example, Blanca, 62, states that while she does not worry about her CD, she does pay attention to her diabetes: “No, I don’t think about [CD]. I think of it as nothing. What I think about is just my sugar, that it doesn’t go up on me.” Blanca’s CD is asymptomatic, so in part this might reflect a pragmatic choice on her part about where to focus her time and attention. On the other hand, there are specific actions she feels she can take to combat her diabetes, such as watching her glucose and controlling her diet. She is able to do something about her diabetes. With CD, often the only viable action is to avoid stress and worry.

Leticia, who is also both diabetic and Chagas-positive, states that “the only thing I do is try to stay *tranquila*. Yes, because I can’t take any type of medicine. Here in the countryside we use sweetened teas to calm down and I can’t because of my sugar (diabetes).” Leticia feels she is unable to avail herself of medicinal teas (usually made from locally available plant sources such as mandarin tree leaves, lemongrass, and the roots of *Alpinia speciosa*, a flower related to ginger) because they are typically prepared with sugar. Thus, trying to stay *tranquila* is the only option left to her. Importantly, her comment expresses the idea that medicinal teas themselves can help a person feel *tranquilidad*.

**Fig 1:**
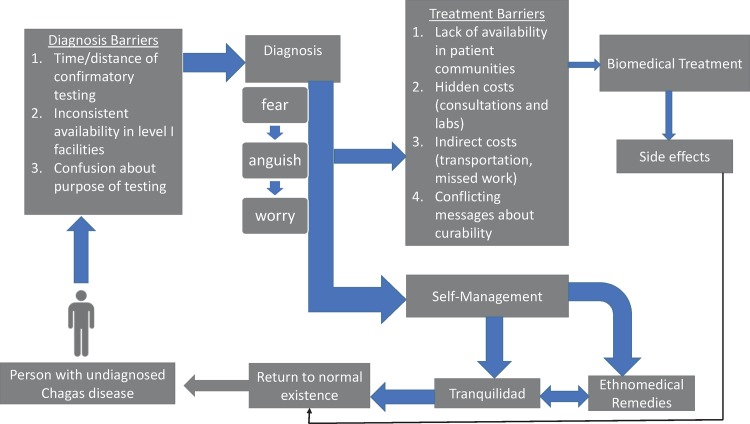
Patient Strategies for Coping with Chagas Disease.

Fig 1 describes the process patients go through following diagnosis of CD. Substantial barriers impede access to biomedical treatment, and patients opt instead to self-manage their CD. Maintaining a feeling of *tranquilidad* helps patients obtain freedom from anxiety and regain a sense of normality in their lives. A return to normalcy could also result from successful biomedical treatment, but the path is much more difficult.

## Discussion

Remaining *tranquilo* helps patients cope with their CD diagnosis and maintain a sense of normality in their lives. Kleinman distinguishes between “disease”: the biological impact of a particular pathogen or disorder, and “illness”: the wide variety of social, emotional, and other impacts experienced by patients in their daily lives[27]. Illness encompasses the ways in which a disease or disorder prevents patients from fulfilling their usual social roles. *Tranquilidad* helps patients manage illness by preventing disruption of daily activities, yet patients perceive it is also critical to preventing a worsening of disease symptoms, which patients feel would be triggered by strong emotions. Respondents indicate *tranquilidad* counteracts the agitation of the heart that often accompanies advanced chronic CD. An individual who is *tranquilo* is in control of worries about impending death or debilitation from CD.

In the social environment, people with CD may suffer from stigmatization because of the disease’s association with poverty[11, 38, 39]. Patients with CD have also faced discrimination, impacting opportunities for employment[9, 40]. Many employers in Bolivia test job candidates for CD. Acting as if CD were “nothing” may help patients cope with or minimize the impact of social stigma.

While it would be tempting to view *tranquilidad* as resignation or fatalism on the part of patients, it is more useful to consider it a coping strategy that enables patients to live with CD, especially given the significant barriers to accessing treatment. These include the distance of providers, hidden costs such as consultations and laboratory analyses, and conflicting messages about the curability of the disease[36]. While past research has identified “fatalism”, defined as a belief that fate rather than individual action determines health outcomes, as a Latino cultural trait and potential barrier to healthcare[41–43], this assumption has come under scrutiny[44]. For instance, Flórez et al. note that Dominican women’s attitudes about breast cancer encompass a complex blend of beliefs which stress both the role of destiny and the impact of individual agency[45]. In other contexts, attitudes which on the surface appear to be “fatalism” could really reflect patient encounters with structural barriers. Drew and Schoenberg found that delays in seeking treatment for cancer patients in eastern Kentucky were not merely a product of fatalism, but often represented a pragmatic response to difficulties in accessing treatment[46].

Other research has examined patient strategies for coping with CD, with similar findings. A study in La Plata, Argentina found that men with CD, while experiencing fear and worry about the disease, went through a process of normalization and acceptance[47]. Similarly, Bolivian migrants in Barcelona with CD viewed the disease as “natural”, often preferring not to obtain treatment as long as no symptoms were present[48]. In Bambuí, Brazil, patients experienced fear and social isolation upon receiving a diagnosis of CD, and felt abandoned by a public health system which focused on vector control over patient treatment. One patient stated it was better not to worry about the disease, since this would worsen its symptoms[49]. The same notion was conveyed by Bolivian patients in this study.

Research has begun to explore the link between CD and emotional health[38, 50]. In a sample of 110 Brazilian patients, 40.9% exhibited depressive symptoms according to the Beck Depression Inventory; those with gastrointestinal complications from CD experienced more depressive symptoms and rated their quality of life lower[50]. In another study, depressive symptoms were correlated with perceptions of quality of life among stroke survivors with CD[51]. In a similar vein, socioeconomic conditions, including low levels of education as was observed in the current study, have been linked to worse progression of chronic CD[52].

In sum, patients in this study feel that excessive anguish or worry over CD would actually aggravate the disease. *Tranquilidad* thus represents a coping strategy which helps patients safeguard their health and return to a semblance of normal functioning. This strategy, along with ethnomedical remedies, permits patients to self-manage their CD, given the difficulties they face in securing biomedical treatment. Current treatment of CD often maintains a narrow, biological focus that disregards patients’ emotional and social well-being. There is a need for more holistic strategies that take into account the socioeconomic conditions and emotional health of patients, enabling them to minimize stigma and return to daily activities[53]. While diagnosis and treatment should be expanded into communities where people with CD live, simultaneous provision of mental health resources and social support may significantly improve adherence to treatment and subsequent outcomes.

### Limitations

Because of the difficulties in confirmatory testing, most patients had only had one test for CD, whereas the World Health Organization recommends both an initial and a confirmatory test. I did not collect reliable data on how many patients were suffering from advanced symptoms of CD. While most were asymptomatic, some had clinically evident CD-related complications, and several suffered from comorbidities including diabetes, age, high blood pressure, and obesity. This study describes a convenience sample of patients whose experiences may not be representative of other patients with CD. Because the author was simultaneously serving as a volunteer at the CMHP, patients may have perceived him as part of the clinic’s staff, which could have influenced some of their responses. The author explained to patients at the beginning of each interview that he was an independent investigator and not a doctor with the clinic, but his positionality may have been unclear to some, especially since most patients were recruited within the clinic. This is essentially a cross-sectional study, so the author was unable to document changes in patients’ coping strategies over time. This is important because the department’s program of free treatment for Chagas disease was in a process of expansion during the study period, so patients may have greater awareness of this program now than they did in 2013 when the investigation took place.

## Supporting Information

S1 Checklist Strobe Checklist. (PDF)

S2 Checklist Primary Quotations. (PDF)

S3 Checklist Primary Quotations Legend. (PDF)
