# Supplementary material for: "I Cannot Be Worried": Living with Chagas Disease in Tropical Bolivia
Source: PLoS Negl Trop Dis. 2017 Jan 18;11(1):e0005251. doi: 10.1371/journal.pntd.0005251 (PMC5242422; doi:10.1371/journal.pntd.0005251)
Supplement: S2 Checklist — (PDF) [file pntd.0005251.s002.pdf]

“I Cannot Be Worried”:

Living with Chagas Disease in Tropical Bolivia

Forsyth, Colin J. **PLOS Neglected Tropical Diseases**

Supporting Information File:
Quotations Pertaining to Tranquilidad

Report: 54 quotation(s) for “tranquilidad”

HU: Chagas qualitative analysis
File: [C:\Users\Colin\Documents\Dissertation\Chagas qualitative analysis.hpr7]
Edited by: Super
Date/Time: 2014-06-08 21:28:51

Mode: quotation list names and references

Quotation-Filter: All

tranquilidad

P 2: Interview 1-02.docx - 2:2 [No se puede estar tranquila] (21:21) (Super)
Codes: [tranquilidad - Family: Ethnomedical domain]
No memos

No se puede estar tranquila

P 2: Interview 1-02.docx - 2:7 [A mi, me agravó, no hace mucho..] (34:34) (Super)
Codes: [tranquilidad - Family: Ethnomedical domain]
No memos

A mi, me agravó, no hace mucho, pero fue mucha tensión. Mucho estrés. Mucho estrés. Eso.

P 2: Interview 1-02.docx - 2:10 [CJF: Como de malhumor? Sí, sí,..] (40:41) (Super)
Codes: [tranquilidad - Family: Ethnomedical domain]
No memos

CJF: Como de malhumor?

Sí, sí, eso. Nada está bien. Pero es uno no más que está mal. Y uno cree que todos están. Así que, eso. Provoca muchas cosas. Sí, provoca muchas cosas. Que a mi, la enfermedad, la marcapaso me separó de mi esposo. Hace 12 años. Este, cuando me pusieron ya marcapaso, el dijo, bueno, ya te vas a morir, mejor me aparto. Pensó que yo no iba a poder hacer nada. Y no fue así, porque yo sigo haciendo mis cosas. Tranquila. Yo costuro, yo hago horneado, yo cazo, yo siembro, yo, todo lo hago como siempre. El salió perdiendo porque el está mal, y está lejos y sin sosten. Bueno, pues, nos abandonó, a mi hija y a mi hijo. Ahora ya es un viejito ya.

P 2: Interview 1-02.docx - 2:11 [CJF: Asi que, cree usted que l..] (44:45) (Super)
Codes: [tranquilidad - Family: Ethnomedical domain]
No memos

CJF: Asi que, cree usted que la pelea con su esposo agravo su Chagas?

Si, Si. Contribuia en, mucho mas, porque cuando yo me enojaba, mas me sentia mal. Me desmayaba. Entonces, era malo.

P 2: Interview 1-02.docx - 2:16 [Cuando me enojo, me hace lo mi..] (51:51) (Super)
Codes: [tranquilidad - Family: Ethnomedical domain]
No memos

Cuando me enojo, me hace lo mismo. Cuando me enojo. No puedo enojarme, no puedo hacer__________. (Rie). No puedo, emocionarme digamos. Cualquier cosa evito, si. Fiesta, todo eso, no puedo estar. A veces me enojo, me alegro mucho, y todo eso hace daño. Hace dano, asi que hay que estar no mas normal.

P 2: Interview 1-02.docx - 2:17 [La Chagas... Para curar? Bueno. ..] (54:56) (Super)
Codes: [tranquilidad - Family: Ethnomedical domain]
No memos

La Chagas… Para curar? Bueno.

CJF: O para manejarlo, tratarlo.

A tratarlo. Lo primero es estar tranquila, no. Estar tranquila. No pensar en que uno está enfermo. Procurar de, este, estar ocupada la mente de cualquier otra cosa, no? Porque si uno dice, por ejemplo cuando me pusieron el marcapaso, yo estaba pendiente. Que no haga nada, que esto, y que esto, que no se agache que no se que. Pero este, me________ ir con el marcapaso mas luego. Trate yo tambien de olvidar. Trato de olvidar. Hay veces que ni recuerdo que tengo. No tengo. Cuando me preguntan, so tengo. Pero ya, yo me saqué eso de mi mente. Sacar la, la posibilidad de no acordarse de que uno tiene esa enfermedad. Porque si uno haga dependiente de la enfermedad, mas se hace pero. Mas. Mas estresa, mas, mas.

P 6: Interview 1-06.docx - 6:5 [La verdad mire, yo no lo tomo ..] (39:39) (Super)
Codes: [tranquilidad - Family: Ethnomedical domain] [treatment of Chagas - Family: Biomedical domain]
No memos

La verdad mire, yo no lo tomo en cuenta. Y solo tomo para la presion. No es mas… Es como si no lo tuviera. No lo tomo en cuenta. Por que nunca me he hecho un tratamiento. Tampoco no se a que grado estara, no?

P 6: Interview 1-06.docx - 6:7 [CJF: con la cascara de la mand..] (47:54) (Super)
Codes: [remedies-heart - Family: Ethnomedical domain] [tranquilidad - Family: Ethnomedical domain]
No memos

CJF: con la cascara de la mandarina me dijo?

El cogollito de la hoja. Paja cedron. Es como un pasto.

CJF: Y la flor de la colonia con el cogollito de la mandarina? De eso se hace un te?

Si, un te. De todo junto, se hace un te.

CJF: Cuantas veces habria que tomar eso?

Una vez, depende de cómo uno siente, de la fatiga que uno siente. Es tomar una vez o dos veces.

CJF: La idea es sacar la fatiga?

A veces uno siente desesperado. Y eso calma

P 8: Interview 1-08.docx - 8:2 [No, no pienso yo en eso. No pi..] (22:26) (Super)
Codes: [tranquilidad - Family: Ethnomedical domain]
No memos

> No, no pienso yo en eso. No pienso en esa enfermedad. Yo pienso que es nada, pues. Lo que yo pienso es mi azucar no mas, que no se me suba.
>
> CJF: Tiene diabetes tambien?

Pero no me sube mucho. Ahorita es por la preocupación, pero no es porque yo no me cuide.

P 8: Interview 1-08.docx - 8:3 [Tengo. Peor cuando mi nietita ..] (30:34) (Super)
Codes: [emotional] [tranquilidad - Family: Ethnomedical domain]
No memos

> Tengo. Peor cuando mi nietita se quemo. Con un termos con agua que le salio la tapa cuando estabamos desayunando. Y entonces me azucar se subio. Antes estaba en 80, 100 no mas. Ahora con la preocupación que tengo, se sube pues.

> CJF: Ud cree tambien que le agrava el Chagas cuando se preocupa?

No, pero si, porque en ese tiempo, cuando me sacaron Chagas, yo tenia mi marido. Yo soy viuda. Tenia mi marido, era cholero. A veces yo digo que por eso me pronuncio el Chagas y el diabetes.

P 8: Interview 1-08.docx - 8:21 [A veces se me suba el azucar. ..] (181:183) (Super)
Codes: [emotional] [tranquilidad - Family: Ethnomedical domain]
No memos

> A veces se me suba el azucar. No es porque yo no me cuide. A veces es para una preocupación que yo tengo. A veces no es para la comida. A veces es para una preocupación que uno tiene. Yo, mi hijo estaba sin trabajo, el menor. Pero ya tiene trabajo gracias a Dios, aunque es lejos, pero tiene. Y es una preocupación menos. Mi hija enferma. Quemada. Y sin plata.

Por eso con el Chagas, yo digo que no tengo. Y no tengo.

P11: Interview 1-11.docx - 11:8 [CJF: Se agrava mas su corazon ..] (104:105) (Super)
Codes: [tranquilidad - Family: Ethnomedical domain]
No memos

CJF: Se agrava mas su corazon si esta preocupada?

Si, me han dicho que no tengo que tener pena. No hay que preocupar dice. Y si alguien estan pelando asi, o hay algun problemita, tenes que apartar me dice. Por que si no te va a fatigar. Para estar tranquilita.

P18: Interview 1-18.docx - 18:2 [Hasta el momento siento que no..] (22:22) (Super)
Codes: [cansancio - Family: symptoms] [tranquilidad - Family: Ethnomedical domain]
No memos

Hasta el momento siento que no tengo Chagas. Tranquila, que no siento fatiga en el corazon, yo pienso de que, de que no tengo ese enfermedad, no? Asi que, me siento tranquila porque otros dicen que tienen Chagas y le fatiga al corazon. Y yo me siento bien hasta el momento, el corazon, y solo que tengo es gastritis.

P21: Interview 2-01.docx - 21:4 [¿Cuándo le dijeron a Ud. que t..] (46:48) (Super)
Codes: [tranquilidad - Family: Ethnomedical domain]
No memos

¿Cuándo le dijeron a Ud. que tiene la enfermedad de Chagas? ¿Cómo se sintió Ud. en ese momento?

Normal. No me preocupe.

P21: Interview 2-01.docx - 21:6 [Pero preocupada que de verdad ..] (58:58) (Super)
Codes: [tranquilidad - Family: Ethnomedical domain] [treatment-avoidance]
No memos

Pero preocupada que de verdad que si yo tengo el Chagas? No. Si se que lo tengo. Pero preocupada no. Si, pero debo preocuparme. Porque me habian citado para ir al doctor en Portachuelo para hacerme la charla de Chagas. Dije voy? Mejor no voy. El doctor me va a insistir y me voy a preocupar, y voy a sentir que estoy enfermo. Pero ahora estoy tranquila. Pero es una cosa de que debemos preocuparnos, porque tarde o temprano, debemos preocuparnos.

P21: Interview 2-01.docx - 21:8 [Esas son las hierbas que nosot..] (80:80) (Super)
Codes: [tranquilidad - Family: Ethnomedical domain]
No memos

Esas son las hierbas que nosotros aca en el campo utilizamos para decir, me duele el corazon, pero uno no sabe de que, no? Tal vez sera emocion, sera alegria, sera tristeza no se. Entonces, hacemos, ponemos unas hierbitas y nos hacemos te. Nos sentimos tranquilizadas.

P22: Interview 2-02.docx - 22:7 [Bueno, a veces mi corazon, pal..] (81:81) (Super)
Codes: [tranquilidad - Family: Ethnomedical domain]
No memos

Bueno, a veces mi corazon, palpita rapido asi. Como si se acelerara. Siempre el dolor de cabeza, pero sera por el azucar que se sube mucho. Todo lo demas normal, sera, porque tenemos tambien que tranquilizarnos, porque tambien si nos ponemos nosotros mal, es otro mal al mismo tiempo.

P22: Interview 2-02.docx - 22:8 [No, porque que mas nos queda? ..] (85:85) (Super)
Codes: [tranquilidad - Family: Ethnomedical domain]
No memos

No, porque que mas nos queda? Hay que resolverlo ya a lo que tenemos, no? Tenemos que poner nuestra parte, tranquilizarnos. Digamos, si aceptar que tenemos esa enfermedad que tenemos que mejorar,

P22: Interview 2-02.docx - 22:10 [CJF: Cuando le vienen esas pal..] (103:109) (Super)

Codes: [ethnomedical - Family: Ethnomedical domain] [tranquilidad - Family: Ethnomedical domain]
No memos

CJF: Cuando le vienen esas palpitaciones o esa corriente, Ud toma algo?

Bueno, lo unico que hago es tratar de tranquilizarme. Si, porque no puedo tomar ningun medicamento, aca en el campo utilizamos un agua endulzada para tranquilizarse y no puedo por motivo de mi azucar. El unico que agarro es irme ante mi mama, mi hermana, para que pase ese rato que le llega mal a uno, no?

CJF: No toma te, ningun remedio casero?

Nada, por motivo del azucar. Lo unico que estoy tomando fue aspirina que me receto el medico.

P24: Interview 2-04.docx - 24:8 [Yo en realidad, como cualquier..] (47:47) (Super)
Codes: [mortality - Family: symptoms] [tranquilidad - Family: Ethnomedical domain]
No memos

Yo en realidad, como cualquier persona, hay muchas veces que te preocupa, no? Uno, por el trabajo que haces. Tienes un pensamiento que cualquier momento te puedes quedar muerto. Y el trabajo que estas haciendo, a lo mejor puede quedar inútil. Pero si pensas bien, tienes por detrás los hijos. O sea la familia. Entonces, uno lo deja en manos de Dios. Y asi, si te pasa algo pues, ni modo. Tampoco vas a amargar la vida simplemente porque te han diagnosticado. Yo se que estoy seguro que tengo esta enfermedad, porque vengo de esa zona donde habia bastantes vinchucas. Entonces yo no puedo decir, o no tengo, porque so yo vivi entre medio de los insectos, entonces, tengo que aceptarlo. Para mi, me siento tranquilo. Sigo adelante con el mismo pensamiento de seguir trabajando, para hacer algo para mis hijos, para mi familia entera. Somos 8 hermanos… y algun dia, si pasa algo, que la familia sepa pues. Porque generalmente pasa algo y luego vienen los procesos que fue esa cosa, otra cosa. Pues a lo mejor si cayo, cayo. Eso, para mi, sigo tranquilo, con las ganas de salir adelante.

P25: Interview 2-05.docx - 25:1 [10) ¿Cuándo le dijeron a Ud. q..] (40:42) (Super)
Codes: [tranquilidad - Family: Ethnomedical domain]
No memos

10) ¿Cuándo le dijeron a Ud. que tiene la enfermedad de Chagas? ¿Cómo se sintió Ud. en ese momento?

Yo creo que normal, porque ya lo he conocido. No mucha preocupación.

P26: Interview 2-06.doc - 26:5 [Y, me puse yo mal. Me senti ma..] (45:49) (Super)
Codes: [faith] [tranquilidad - Family: Ethnomedical domain]
No memos

Y, me puse yo mal. Me senti mal, me afecto mucho saber que yo tenia esa enfermedad. Yo no comia, yo no dormia. Me sentia yo mal, pensando que yo me iba a morir. Y después, que hice? Decidirme pedir a Dios y la Virgen que me quiten ese pensamiento. Me lo quito y me olvide que tenia esa enfermedad.

CJF: Usted se sentia muy preocupada sobre todo.

Si, me afecto mucho. Asi que ya, ya les pedi al Senor y a la Virgen que me quite ese pensamiento. Y me lo quite y me olvido yo que tengo Chagas.

P26: Interview 2-06.doc - 26:16 [Bueno, lo unico que yo le dije..] (109:109) (Super)
Codes: [tranquilidad - Family: Ethnomedical domain]
No memos

Bueno, lo unico que yo le dije es que yo me preocupe mucho al saber que tenia esa Chagas. Mucho me preocupe. Pero después no. Ya me olvide. Tranquilize, conformarse pues con la enfermedad.

P27: Interview 2-07.docx - 27:5 [Bien asustada. Me dijeron que ..] (46:46) (Super)
Codes: [emotional] [tranquilidad - Family: Ethnomedical domain]
No memos

Bien asustada. Me dijeron que no, no, no. Esta dormida. Entonces ya me calme. Mucho yo preocupada, no dormia pues.

P29: Interview 2-10.docx - 29:3 [Eso tengo de estar tranquila. ..] (43:47) (Super)
Codes: [tranquilidad - Family: Ethnomedical domain]
No memos

Eso tengo de estar tranquila. Estoy media muerta pero no preocupada. (rie)

CJF: Hasta ahora no se siente preocupada?

No. Tal vez porque yo estoy controlando. Que con ese control, estoy bien no mas.

P31: Interview 2-12.docx - 31:5 [¿Cuándo le dijeron a Ud. que t..] (42:44) (Super)
Codes: [tranquilidad - Family: Ethnomedical domain]
No memos

¿Cuándo le dijeron a Ud. que tiene la enfermedad de Chagas? ¿Cómo se sintió Ud. en ese momento?

Mire yo la creia y no la creia, pero tanto lo creia, y pues, tranquila porque para que nos vamos a afligir? Tranquila, de algo uno tiene que morir, no?

P37: Interview 2-18.docx - 37:3 [No me asuste, nada, ahorita en..] (44:44) (Super)
Codes: [tranquilidad - Family: Ethnomedical domain]
No memos

No me asuste, nada, ahorita en Santa Cruz es una enfermedad tan comun. Si se detecta a tiempo y se trata, no tiene ningunas consecuencias graves. Tal vez es por eso que no lo di mucha importancia.

P38: Interview 2-19.docx - 38:2 [CJF: Actualmente se siente pre..] (42:44) (Super)
Codes: [faith] [tranquilidad - Family: Ethnomedical domain]
No memos

CJF: Actualmente se siente preocupada?

Muy poco. Porque solo Dios sabe. El es que nos protege y nos, este, de todo.

P40: Interview 2-21.docx - 40:3 [Como siempre escuchaba que ten..] (41:41) (Super)
Codes: [curability] [tranquilidad - Family: Ethnomedical domain] [treatment-biomedical]
No memos

Como siempre escuchaba que tenia Chagas y por eso se murio de un enfarto, no?… Es un impacto, no, sentir que uno tiene esa enfermedad. Despues el doctor me dijo no hay cura, no mas Usted tiene que cuidarse, este de BEJE. Despues aca tampoco, no me dio nada, dijo que estaba dormido. Y bueno, que quedara yo tranquila. Dije doctor como es que esta dormido, bueno dijo no tenga pena, es hasta alla no mas dijeron en CENETROP. No tenga pena, que esta dormida. Ya aca tambien el doctor dijo no tenga pena. Esta dormida.

P41: Interview 2-22.docx - 41:2 [No se, ahorita yo, lo tomo si,..] (36:36) (Super)
Codes: [tranquilidad - Family: Ethnomedical domain]
No memos

No se, ahorita yo, lo tomo si, que fuera no enferma no? No tengo preocupación ya.

P41: Interview 2-22.docx - 41:4 [U, yo me sentia mal. Mi madre ..] (43:43) (Super)
Codes: [emotional] [tranquilidad - Family: Ethnomedical domain]
No memos

U, yo me sentia mal. Mi madre vendia antes en el mercado, en el comedor donde hay comida, u yo lloraba alli, me desespere. Era lo mas triste, no, estar con Chagas, porque me dijeron que era peligroso, que uno se moria. Pero el dia después me dijo una amiga, no, no te preocupes porque todingos tenemos Chagas. Yo tengo Chagas y mira como me veo. De eso yo quede medio tranquila. Yo pense que me iba a morir, que tengo Chagas, y mis hijos estan chicos,

P41: Interview 2-22.docx - 41:8 [Ya después de que me hablaron ..] (81:81) (Super)
Codes: [tranquilidad - Family: Ethnomedical domain]
No memos

Ya después de que me hablaron asi de Chagas, ya no lo pongo atención. Se me ha olvidado creo (rie).

P41: Interview 2-22.docx - 41:11 [Manzanilla, para tranquilizars..] (91:91) (Super)
Codes: [remedies-heart - Family: Ethnomedical domain] [tranquilidad - Family: Ethnomedical domain]
No memos

Manzanilla, para tranquilizarse.

P42: Interview 2-23.docx - 42:8 [Lo primero, sabes que es? La t..] (93:93) (Super)
Codes: [tranquilidad - Family: Ethnomedical domain]
No memos

Lo primero, sabes que es? La tranquilidad de uno. Eso. Porque asi no afecta mucho al corazon, se fatiga uno, no ves? Eso de la tranquilidad es lo mas, porque hay veces que uno se fatiga, no? Eso es lo mas, este.

P42: Interview 2-23.docx - 42:10 [Ayudarlos a que se mantengan a..] (102:102) (Super)
Codes: [tranquilidad - Family: Ethnomedical domain] [vicios]
No memos

Ayudarlos a que se mantengan asi tranquilos, y la cosa es tambien lo que beben, no ves? Eso empeora.

La bebida, no ves? Y ahorita que esta esa coca, que mastican, eso tambien.

P43: Interview 2-24.docx - 43:6 [10) ¿Cuándo le dijeron a Ud. q..] (72:74) (Super)
Codes: [tranquilidad - Family: Ethnomedical domain]
No memos

10) ¿Cuándo le dijeron a Ud. que tiene la enfermedad de Chagas? ¿Cómo se sintió Ud. en ese momento?

Tranquilo. Porque hemos llegado a este mundo, y soy conciente de que tenemos que irnos. No es para toda la vida. Tranquilo. Tranquilo.

P44: Interview 2-25.docx - 44:4 [Cuando la persona agarra la en..] (51:51) (Super)
Codes: [emotional] [tranquilidad - Family: Ethnomedical domain]
No memos

Cuando la persona agarra la enfermedad, es muchas veces psicologico. Porque al comienzo se me dio yo lloraba, porque yo vi una senora que se habia muerto, varias del corazon, no? Como me voy a enfermar de eso, dije, si yo estaba debajo de 30 anos. Entonces dije bah, y vivi mi vida y me olvide de eso. No, ahorita es que no la tenga. Ni el azucar. El unico malo que me hace sentir son los huesos. Porque el otro no me duele, no me hace nada. El Chagas no, ya me olvide de eso. Se que lo tenia, de repente no tenia, después lo tenia… A, dije. De algo va a morir uno. Es lo que algunos dicen.

P46: Interview 2-27.docx - 46:3 [Nada, me senti tranquila. Igua..] (49:49) (Super)
Codes: [tranquilidad - Family: Ethnomedical domain]
No memos

Nada, me senti tranquila. Igual uno va a morir de algo. Esperar que llegue no mas.

P48: Interview 2-29.docx - 48:3 [No me afecta nada, por eso no ..] (56:56) (Super)
Codes: [tranquilidad - Family: Ethnomedical domain] [treatment-avoidance]
No memos

No me afecta nada, por eso no he ido. Tranquilo no mas.

P49: Interview 2-30.docx - 49:3 [Tranquilo no mas, porque dicen..] (45:45) (Super)
Codes: [mortality - Family: symptoms] [tranquilidad - Family: Ethnomedical domain]
No memos

Tranquilo no mas, porque dicen que no tiene mucho dolor el Chagas. Mata no mas, dice. (Rie). Asi han

dicho.

P51: Interview 2-32.docx - 51:6 [No es que no cumple en mi casa..] (71:71) (Super)
Codes: [tranquilidad - Family: Ethnomedical domain]
No memos

No es que no cumple en mi casa. Pero yo trato de cumplir. O sea, parece que es el esfuerzo de cumplir en la casa, pero pese a eso, no doy conformidad. Y ese es el malestar a veces. Es que me preocupa o me molesta. O a una vez las dos cosas. Porque cuando yo estoy tranquila, yo no siento nada. Cuando estoy tranquila.

P52: Interview 2-33.docx - 52:2 [Normal no mas, no sentia que m..] (48:48) (Super)
Codes: [tranquilidad - Family: Ethnomedical domain]
No memos

Normal no mas, no sentia que me voy a morir. Tranquila. Ni modo que hay que hacerlo.

P56: Interview 2-37.docx - 56:3 [No lo tome nada grave. Tranqui..] (42:48) (Super)
Codes: [tranquilidad - Family: Ethnomedical domain]
No memos

> No lo tome nada grave. Tranquila no mas, porque, mi mama tambien tenia Chagas pero ella ya ha fallecido.
>
> CJF: Ella fallecio de Chagas?
>
> Chagas porque le dio un ataque. Le iban a poner marcapasos, y de un ataque se murio. Faltaban dos dias para que le pongan el marcapasos.

Porque no he sentido mal, no he ido a buscar tratamiento. Mi esposo dijo hay que hacerse ver, pero no lo he hecho tampoco porque no senti nada grave.

P57: Interview 2-38.docx - 57:2 [Bien, no senti nada. No me afe..] (41:41) (Super)
Codes: [tranquilidad - Family: Ethnomedical domain]
No memos

Bien, no senti nada. No me afecto, pense que era una enfermdad que, bueno, como cualquier cosa asi

no mas. No dije yo, ay, que eso me va a matar o algo asi, no. Bueno, tiene Chagas, tranquila no mas. Como si nada. Alli no mas quedo. Bueno, estoy fatigadita asi pero nunca penso que puede ser del Chagas, no? Nunca. Nunca se me ha venido a la mente, ay sera por el Chagas o porque tengo Chagas. No. Yo digo porque me fatigo o porque siento esto aquí? No es mas. O sea, sigo haciendo mis cosas, y me olvido. Pero cuando ya me echo en la noche, y quiero ponerme a este lado, siento fatiga. Siento. Me echo asi de espalda, siento fatiga. Asi que, mas duermo a este lado. Bueno, yo digo, sera del Chagas o sera del corazon?

P57: Interview 2-38.docx - 57:6 [Conoce la colonia? Eso, yo me ..] (67:77) (Super)
Codes: [remedies-heart - Family: Ethnomedical domain] [tranquilidad - Family: Ethnomedical domain]
No memos

Conoce la colonia? Eso, yo me hago un te de colonia. Un te de mandarino. De cogollito de mandarino.

CJF: Esos son para la fatiga?

O sea, yo siento que tomando esos tecitos me tranquilizo, estoy un poco mas tranquila.

CJF: Ayudan cuando esta estresada tambien?

Me ayudan bastante.

Hay otra plantita, el toronjil tambien.

P57: Interview 2-38.docx - 57:9 [Nunca me preocupe de eso. Nunc..] (95:95) (Super)
Codes: [tranquilidad - Family: Ethnomedical domain] [treatment-avoidance]
No memos

Nunca me preocupe de eso. Nunca. Supe que tenia eso, bueno, me quede asi no mas, tranquila. O sea, yo siento que no es una enfermedad para que uno, bueno, este alli, no? Pensando que estoy con esto. No, no. Pero entonces si yo estaba con eso mas fluido, entonces ya pongo mas interes. Y digo bueno, voy a entrar en tratamiento, voy a seguirlo,

P58: Interview 2-39.docx - 58:4 [CJF: Al principio sentia un po..] (43:49) (Super)
Codes: [tranquilidad - Family: Ethnomedical domain]
No memos

CJF: Al principio sentia un poco preocupado?

Mal. Despues, sabes que Colin? Me senti transuilo, a pesar de lo que tenia, no le di mucha importancia. Eso fue lo bueno.

CJF: Que le ayudo a sentir mas tranquilo?

De estar en el lugar. Como decirle aquí trabajo con Ustedes, de alguna cosa, de limpiar o acomodar, me siento contento ya asi. Y bien, uno ya se familiariza ya. (Hace 8 anos)

P61: Interview 2-42.docx - 61:2 [Algo preocupante, porque es su..] (45:45) (Super)
Codes: [diagnosis - Family: Biomedical domain] [disease model] [em-time, symptom onset] [tranquilidad - Family: Ethnomedical domain]
No memos

Algo preocupante, porque es su salud de uno, no? Cuando me detectaron esa vez, no me dijeron para alarmarme asi. Tiene Chagas, esta si. Tiene Chagas, pero no para preocuparse, me dijo. Pero no me afecto mucho como cuando me entere que tenia diabetes. Eso fue terrible para mi. No se, porque yo senti que el diabetes era para morirse digamos. Que ya no tenia mucha vida ya. El Chagas no me afecto mucho, la verdad. Porque como me dijeron alla, no esta muy avanzado. Esta recien, eso se puede controlar.

P61: Interview 2-42.docx - 61:5 [Como no me alarmaron no me pre..] (77:77) (Super)
Codes: [tranquilidad - Family: Ethnomedical domain]
No memos

Como no me alarmaron no me preocupe. Tranquila.

P62: Interview 2-43.docx - 62:2 [Bueno, es una enfermedad del c..] (41:41) (Super)
Codes: [disease model] [tranquilidad - Family: Ethnomedical domain]
No memos

Bueno, es una enfermedad del corazon, pero cuando uno se trata, entra un tratamiento, tranquilo, para mi es tranquilo.

P62: Interview 2-43.docx - 62:3 [No, no me preocupe nada, como ..] (46:46) (Super)
Codes: [tranquilidad - Family: Ethnomedical domain]
No memos

No, no me preocupe nada, como siempre hago, como tengo diabetes tambien, no me preocupo. No tengo esa preocupación. Para mi es igual si estoy con diabetes o estoy con Chagas, es igual para mi. Yo trato de estar tranquila, no acordarme, como si no tuviera nada. Hay personas que, si le detectan alguna enfermedad, se preocupan. Yo no. No estoy de esas personas. Tranquila.

P64: Interview 2-45.docx - 64:3 [Bueno, yo lo tome como cualqui..] (44:44) (Super)
Codes: [tranquilidad - Family: Ethnomedical domain]
No memos

Bueno, yo lo tome como cualquier enfermedad. Para no deprimirse, no? Asi no mas lo tomo. Porque me dijeron que no tenia que preocuparme. De la enfermedad que tenia asi. Muy rapido yo se, digamos, que empeora. O sentarse a pensar en la enfermedad. Yo toda la vida trabaje, y en ese me mantengo normal. Es como si no tenia ninguna cosa, enfermedad. Trabajo, trabajo y trabajo, y no se si estoy enferma ni nada. Asi me gusta.

P65: Interview 2-46.docx - 65:2 [No se, yo no siento nada. Yo n..] (40:40) (Super)
Codes: [asymptomatic] [tranquilidad - Family: Ethnomedical domain]
No memos

No se, yo no siento nada. Yo no siento mareo, no siento dolor. A veces la circulación, esto me duele, pero voy al Centro, ya me dan medicamentos y punto. Quedo bien. Pero después, no me fatigo. No tengo problemas de presion. Nada. Tranquila.

P65: Interview 2-46.docx - 65:5 [Yo no quiero preocuparme. Sabe..] (81:81) (Super)
Codes: [tranquilidad - Family: Ethnomedical domain] [treatment-avoidance]
No memos

Yo no quiero preocuparme. Sabe por que? Por mi esposo. El no hay quien lo atienda. Imaginese que me diga Usted tiene Chagas, esta avanzando, ahora manana voy a morir. Eso voy a pensar, no ves? Voy a decir ay, esta avanzando mi Chagas, voy a morir en cualquier momento. Que va a ser de mis hijos. Que va a ser de mi marido. En fin mis hijos son siete, son mayores. Y si me dice esta avanzando su Chagas, yo me voy a preocupar. Voy a pensar, ahora manana voy a morir. Tal vez me voy a dormir, manana ya voy a estar muerta. Que va a ser de mi esposo, quien lo va – mi hijo. Entonces por eso no quiero.
