# Supplementary material for: "I Cannot Be Worried": Living with Chagas Disease in Tropical Bolivia
Source: PLoS Negl Trop Dis. 2017 Jan 18;11(1):e0005251. doi: 10.1371/journal.pntd.0005251 (PMC5242422; doi:10.1371/journal.pntd.0005251)
Supplement: S3 Checklist — (PDF) [file pntd.0005251.s003.pdf]

# Supporting Information Legend

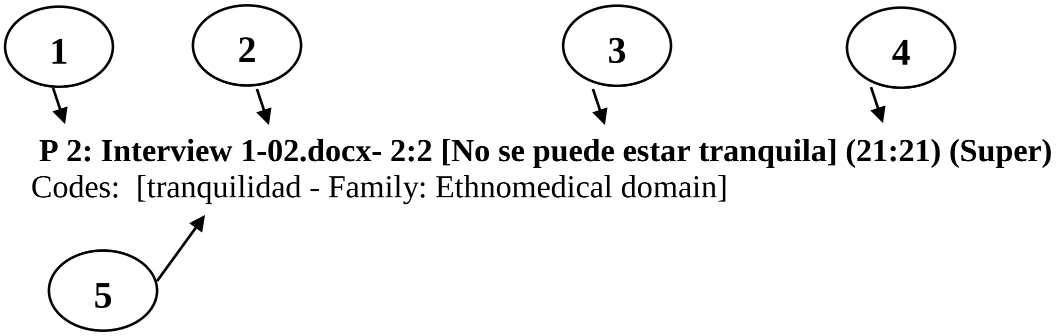


1) **P2=patient ID**

2) **Interview 1-02.docx-2:2: Transcript document #**

**3) [No se puede estar tranquila]: Leading words of quotation**

**4) (21:21) Demarcation of the lines where the quotation occurs within the interview transcript.**

**5)** [tranquilidad - Family: Ethnomedical domain]: This indicates the coding structure (this quote was assigned the code “tranquilidad”, which is under the “ethnomedical domain” heading).
